# Association of over-the-counter mouthwash use with markers of nitric oxide metabolism, inflammation, and endothelial function—a cross-sectional study

**DOI:** 10.3389/froh.2025.1488286

**Published:** 2025-01-27

**Authors:** Kai Guo, Kaumudi Joshipura, Karina Ricart, Rakesh P. Patel, Barbara A. Gower, Oelisoa Mireille Andriankaja, Evangelia Morou-Bermudez

**Affiliations:** ^1^ Surgical Science Department, School of Dental Medicine, University of Puerto Rico, San Juan, PR, United States; ^2^ School of Public Health, Ahmedabad University, Ahmedabad, India; ^3^ Department of Pathology, University of Alabama at Birmingham, Birmingham, AL, United States; ^4^ Department of Nutrition Sciences, Division of Physiology & Metabolism, School of Medicine, University of Alabama at Birmingham, Birmingham, AL, United States; ^5^ Center for Oral Health Research (COHR), College of Dentistry, University of Kentucky, Lexington, KY, United States

**Keywords:** over-the-counter (OTC) mouthwash, inflammatory biomarkers, endothelial function, nitric oxide (NO), nitrite (NO_2_), nitrate (NO_3_)

## Abstract

**Introduction:**

Regular use of mouthwash can disrupt nitrate reduction by oral bacteria and may affect systemic nitric oxide (NO) levels, which are important for inflammation and endothelial function. We aim to assess the association between over-the-counter (OTC) mouthwash use and nitrate/nitrite, markers of inflammation (IL-6, TNF-α, CRP) and endothelial function (sICAM-1, sVCAM-1) in serum and saliva, and to assess the relationship between nitrate/nitrite levels and these biomarkers, as well as how OTC mouthwash modulated this relationship. We hypothesize that nitrates/nitrites are associated with these biomarkers, and that their associations would vary with the frequency of mouthwash use.

**Method:**

Our cross-sectional study used data and specimen from the baseline of the San Juan Overweight Adult Longitudinal Study (SOALS). Robust Gamma regression with log-link function, Spearman correlations and partial correlations adjusted for covariates were used for the analysis.

**Results:**

Using OTC mouthwash twice a day or more was significantly associated with lower serum nitrite levels compared to less frequent use (β = −0.357, 95% CI: −0.650, −0.064), but not with other markers of inflammation and endothelial function. Mouthwash use differentially impacted the relationship between nitrate/nitrite and TNF-α, sICAM-1 and sVCAM-1. Specifically, in the participants who used mouthwash less than twice a day or no use, TNF-α (β = −0.35, 95% CI: −0.52, −0.18), and sICAM-1 (β = −0.21, 95% CI: −0.32, −0.09) were negatively associated with serum nitrite. In the participants who used mouthwash twice a day or more use, TNF-α was positively associated with serum nitrate (β = 3.36, 95% CI: 2.07, 4.65), salivary nitrite (β = 1.04, 95% CI: 0.39, 1.69) and salivary nitrate (β = 0.48, 95% CI: 0.25, 0.71); sICAM-1 was positively associated with serum nitrate (β = 1.58, 95% CI: 0.86, 2.29). In both subgroups of mouthwash users, sVCAM-1 was positively correlated with serum nitrate and salivary nitrate. In addition, sVCAM-1 was positively correlated with serum nitrite in participants who used mouthwash frequently (*ρ*__S_ = 0.18, *p* = 0.045).

**Discussion:**

Regular use of OTC mouthwash was associated with systemic nitric oxide. This raises concerns about its potential effects on the levels of inflammatory and endothelial biomarkers associated with cardiometabolic diseases.

## 1 Introduction

Endothelial cells are involved in several processes including regulation of vascular tone, cell adhesion, smooth muscle proliferation and inflammation (1). Endothelial dysfunction leads to an imbalance of vasodilation and vasoconstriction, elevated reactive oxygen species (ROS) and inflammatory factors, and diminished nitric oxide dependent signaling. Endothelial cell dysfunction, together with systemic and local inflammation are closely related to the development of atherosclerosis, which leads to cardiometabolic diseases (CVD).

During inflammation, endothelial cells become activated and produce cytokines such as interleukin-6 (IL-6), tumor necrosis factor (TNF-α), and upregulate adhesion molecules, including intercellular adhesion molecule-1 (sICAM-1), vascular adhesion molecule-1 (sVCAM-1). These inflammatory cytokines stimulate the hepatic secretion of C-reaction protein (CRP) and other mediators. TNF-α and IL-6 are multifunctional cytokines with multiple biological activities. Many studies have confirmed that TNF-α and IL-6 promote oxidative stress, lead to continuous production of lipid peroxides, produce multiple toxic factors, cause vascular endothelial damage, and interfere with prostaglandin homeostasis (2). sICAM-1 and sVCAM-1 play a critical role in mediating the firm adhesion of leukocytes to vascular endothelial cells in various acute and chronic inflammatory diseases. In addition, C-reactive protein (CRP) is a protein that appears in the acute phase of infectious or non-infectious inflammatory diseases (3). CRP is not just an inflammatory marker but also directly involved in the inflammatory process itself (4): it stimulates monocytes to release inflammatory mediators, including TNF-α, IL-6, and IL-1β, promotes vascular endothelial cells to up-regulate the expression of adhesive factors, such as sICAM-1 and sVCAM-1, and induces endothelial cells to express pro-inflammatory cytokines.

Nitric oxide (NO) is a signaling molecule that regulates inflammation, vascular tone, and insulin sensitivity. It exerts a protective function in the vascular endothelium, reducing levels of inflammation markers and endothelial damage (5, 6). In endothelial cells, NO is synthesized from L-arginine via the endothelial nitric oxide synthase (eNOS). More recent studies have identified an additional source of NO-signaling equivalents from dietary nitrate consumption that is independent of nitric oxide synthase (7–9). Specifically, commensal oral bacteria convert exogenous (dietary) and endogenous (recycled) nitrate (NO_3_) into nitrite (NO_2_). Upon swallowing, nitrite is converted to NO via host pathways in the circulation and tissues. Mouthwash has been promoted to be part of daily oral hygiene routine, hence many people use it regularly, once or even twice a day. However, regular use of mouthwash can disrupt oral nitrate metabolism, affecting systemic NO levels and related biological and clinical outcomes (10).

The potential link between mouthwash use and systemic health has gained increasing interest in recent years, particularly as the COVID-19 pandemic has increased the focus on the role of mouthwash as an oral antiseptic that may be useful in reducing the oral viral load. Some studies highlighted some common systemic conditions that are influenced by mouthwash use, including cardiovascular disease, diabetes, oral cancer, etc (11, 12). Mouthwashes have different modes of action, depending on their active ingredients, concentrations, and how and how often they are used. In the case of over-the-counter (OTC) mouthwash, our previous publications from the San Juan Overweight Adults Longitudinal Study (SOALS) among 945 individuals demonstrated that frequent routine use of OTC mouthwash significantly increased the risk of prediabetes/diabetes and hypertension independently of major confounders (13, 14). Additionally, some studies have shown that the use of mouthwash impacts the levels of nitric oxide metabolites, and reduces serum and salivary levels of CRP, TNF-α, and IL-6 due to its anti-inflammatory effect, sustained bacteriostatic effect and regulation of oral flora balance (15–18). However, most of these studies focused on specific types of mouthwash, including chlorhexidine or mouthwash combinations containing other ingredients (e.g., aspirin or minocycline) rather than OTC mouthwashes; targeted only specific populations (e.g., patients with periodontitis); were short-term clinical trials; and focused only on salivary nitrate and nitrite levels rather than systemic. Studies assessing the association between serum and salivary nitric oxide metabolites and pro-inflammatory markers and markers of endothelial dysfunction are limited, and little is known about how regular use of over-the-counter mouthwash affects these associations. In fact, these elements have never existed independently but have interacted and operated synergistically. The objective of this cross-sectional study, is to utilize the baseline data of SOALS to assess potential associations between OTC mouthwash use and pro-inflammatory (IL-6, TNF-α, CRP) markers, markers of endothelial dysfunction (sICAM-1, sVCAM-1) and nitric oxide metabolites, and to evaluate the relationships between these markers among overweight/obese individuals. We hypothesize that regular use of OTC mouthwash impacts the levels of nitrates/nitrites and inflammatory markers and endothelial function; nitrates/nitrites are associated with inflammatory markers and endothelial function, and the associations vary with the frequency of OTC mouthwash use.

## 2 Materials and methods

### 2.1 Study population

The San Juan Overweight Adults Longitudinal Study (SOALS) is a cohort of non-institutionalized Hispanic adults in Puerto Rico recruited primarily from the San Juan metropolitan area (13). Its recruitment and baseline data collection started in 2011, and the 3-year follow-up began in 2014 and was completed in 2016. Inclusion criteria for SOALS at baseline included (1) age between 40 and 65 years; (2) overweight/obese (body mass index, BMI≥25.0 kg/m^2^); and (3) no physician diagnosis of diabetes or major cardiovascular disease. Additional exclusion criteria included pregnancy, hypoglycemia, congenital heart murmurs or heart disease, heart valve disease, endocarditis, rheumatic fever, bleeding disorders and active dialysis treatment. Informed consent was obtained from all participants prior to performing the study procedures. Interviewer administrated questionnaires (Supplementary File 1) collected information on age, sex, smoking, alcohol intake, frequency of oral hygiene aids including OTC mouthwash use (not limit type/brand), dental treatment and history, anthropometric measurements (NHANES III procedures), physician diagnosed diseases, family medical history, medication use, sleep breathing disorders, time and frequency of physical activity during a typical week, how often participants ate specific food items that were pertinent to diabetes risk, etc. Blood pressure was measured three times following the gold standard Korotkoff auscultatory method. Full-mouth oral exams included probing depth, gingival recession, plaque index, and bleeding on probing (BOP), number of missing teeth and caries (a modified version of the NHANES procedures). In addition, blood samples were drawn at fasting (used in this present study), and at 30-mins, 1-hour and 2-hour after consumption of a glucose drink containing 75 g dextrose. Fasting blood was processed for glucose, insulin, HbA1c and hs-CRP at that time. HOMA-IR was calculated using fasting glucose and insulin. In addition, samples of saliva, plaque and gingival crevicular fluid (GCF) were also collected in SOALS baseline study.

**Figure 1:**
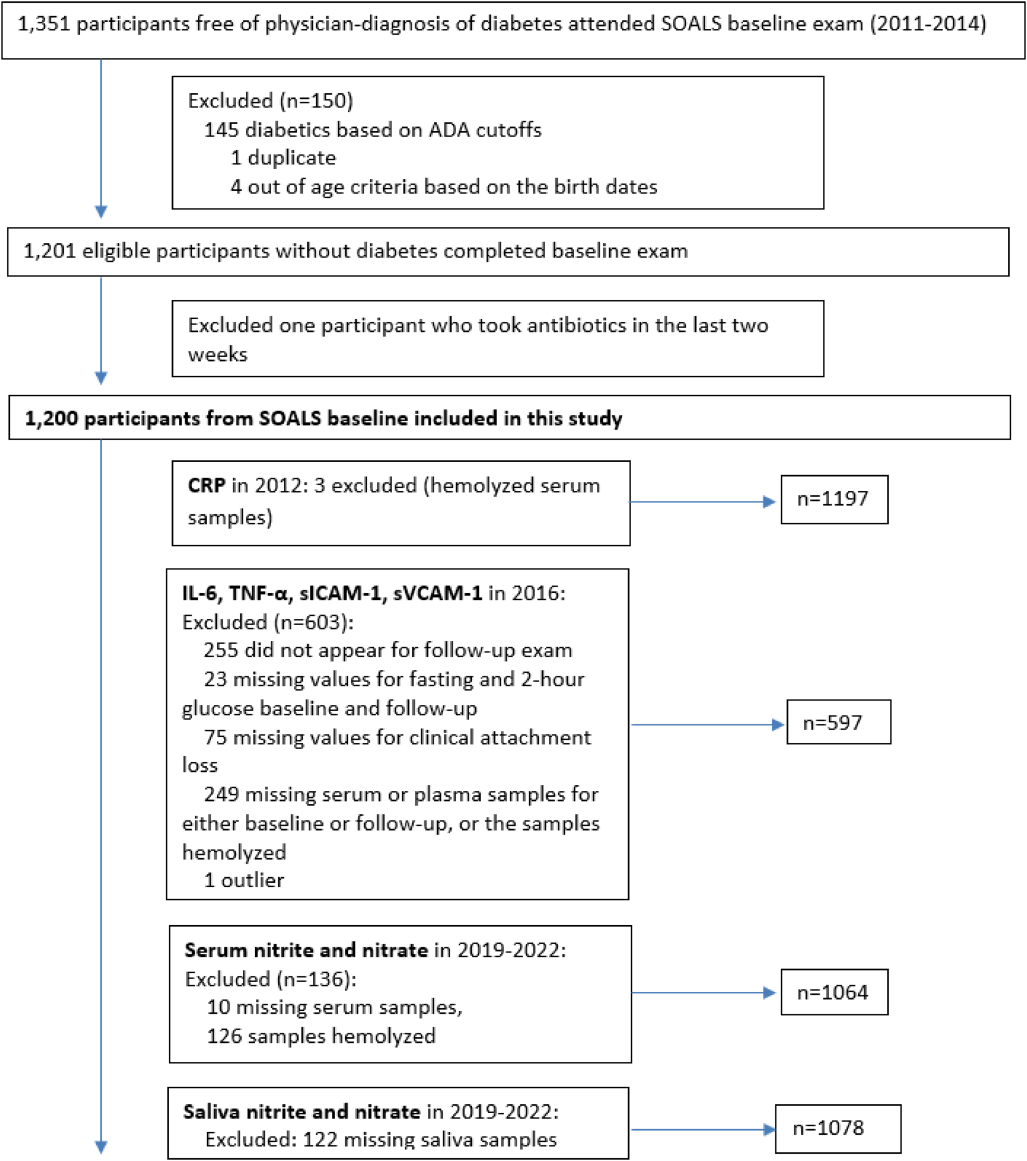
Flow diagram of recruitment of participants from the previous study of San Juan Overweight Adults Longitudinal Study (SOALS).

This current study used biospecimen (fasting serum and saliva), clinical and questionnaire data (e.g., mouthwash use and potential confounders) from SOALS baseline. Of the 1,351 participants enrolled in SOALS baseline study, 4 were excluded because they were out of range for age (confirmed by date of birth), 1 was excluded because we identified that participant came to the SOALS baseline twice, bringing the total number of eligible SOALS participants to 1,346. In this present cross-sectional study, 145 participants with diabetes mellitus (laboratory diagnosis) and one participant on antibiotics were further excluded, resulting in a total of 1,200 participants (Figure 1). The study was approved by the Institutional Review Board of the Office for the Protection of Human Research Subjects at the University of Puerto Rico on February 1, 2019 (UPR Institutional Review Board, IRB#A4840318), and reported in accordance with STROBE guidelines (Supplementary File “STROBE checklist”). All procedures were performed in compliance with the Declaration of Helsinki of 1975, as revised in 2013, and institutional guidelines. Informed consent was obtained from all participants prior to performing the study procedures.

### 2.2 Sample collection and laboratory measurements

Methods for blood and saliva collection in SOALS baseline have been previously described in detail (13, 14, 19). Briefly, SOALS participants were asked to fast for 10 h prior to the study visit. Venous blood samples were drawn at fasting using a standard protocol and silicone-coated sterile blood collection tubes (Becton Dickinson Vacutainer Systems, Franklin Lakes, NJ, USA). Blood was centrifuged at 3,000 rpm for 15 min within 10 min of blood draw to separate RBC from serum/plasma, and EDTA tubes for plasma samples and serum were frozen and stored at −80℃. Unstimulated saliva samples were collected 30 min after consumption of glucose drink containing 75 g dextrose, and were centrifuged (2,600 × g, 15 min at 4℃). Supernatant was aliquoted and stored at −80℃.

Serum high-sensitivity C-reactive protein (hs-CRP) values were assessed in 2012 by the Immuno Reference Laboratory in Puerto Rico, and measurements of serum markers of inflammatory (IL-6, TNF-α) and endothelial dysfunction (sICAM-1 and sVCAM-1) were analyzed and ascertained at the University of Alabama at Birmingham in 2016 (19).

SOALS baseline fasting serum and after-glucose saliva samples were transported in several batches during 17th July 2019 and 24th January 2022 on dry ice to Dr. Rakesh Patel's laboratory at University of Alabama at Birmingham for nitric oxide metabolites (nitrite and nitrate) measurements. Serum samples were thawed on ice in the dark and mixed with ice-cold methanol (1:2 vol:vol) and centrifuged (10,000 × g, 10 min). Serum and saliva nitrite and nitrate were measured by HPLC (high-performance liquid chromatography)-coupled to the Griess assay (Eicom) (20–22), and later calculated by comparison with standard curves (20, 23). Nitrite levels were also measured by triodide reduction to NO and measurement by reaction with ozone using the Sievers^TM^ Nitric Oxide Analyzer NOA 280i instrument (Zysense, NC). The limit of detection (LOD) for serum nitrite was 0.01 µM. 346 (32.52%) of the 1,064 serum nitrite measurements were below the LOD. These measurements were substituted by LOD/2, i.e., 0.005 µM, because the data were highly skewed with geometric standard deviation (GSD) factor of 3.78, which is greater than 3.0 (24). In addition, serum nitrite and nitrate measurements were adjusted for recovery efficiency rate, which corresponded to the percent recovery of nitrite and nitrate after the samples were treated with methanol for protein removal. Saliva samples did not require protein extraction prior to measurement; therefore, salivary nitrite and nitrate values were not adjusted for recovery efficiency rate. Serum nitrite was measured in µM, while serum nitrate, salivary nitrite, and salivary nitrate were measured in mM.

Although laboratory measurements of all the same biomarker were conducted during a time interval (e.g., serum and salivary nitrite and nitrate were measured from 2019 to 2022), standardized laboratory methods and standards were adopted to obtain consistent and reliable results.

### 2.3 Mouthwash use and covariates assessment

The SOALS baseline interviewers-administered questionnaire assessed information on the frequency of use of over-the-counter (OTC) mouthwash. Because the aim of SOALS was to assess the overall risk of using over-the-counter mouthwash, rather than any specific type or brand, the different types of mouthwash were not distinguished. The primary exposure in this present study, frequency of mouthwash use, was categorized as twice or more daily use vs. less frequent use or no use, which is consistent with previous SOALS publications (13, 14), published clinical trials literature (25–27) and advertisements/recommendations suggesting that alcohol-free mouthwash significantly reduces oral bacteria over a period of up to 12 h (28). We refer to mouthwash use twice or more per day as “frequent use” and less frequent or no use as “infrequent use”. Important covariates collected through the SOALS baseline questionnaire included age, sex, smoking status (nonsmoker, former smoker, current smoker), and duration and frequency of physical activity in a typical week. A metabolic equivalent (MET) score was assigned to each activity based on intensity, and the total MET score for each participant was calculated as MET hours/week.

### 2.4 Sample size

Out of a total of 1,200 eligible participants, 126 participants had hemolysis in their fasting serum samples and 10 participants had missing samples, so serum nitrite and nitrate levels were measured for 1,064 participants. Of the 1,200 participants, 122 participants had missing saliva samples, so saliva nitrite and nitrate were measured for 1,078 participants.

Of the 1,200 participants, 1,197 serum hs-CRP values were assessed in 2012 after excluding 3hemolyzed serum samples. 602 participants were excluded from the measurements of serum markers of inflammatory (IL-6, TNF-α) and endothelial dysfunction (sICAM-1 and sVCAM-1), if they did not complete SOALS follow-up, or lacked key data for SOALS (smoking status, periodontal parameters, and glucose levels), or had missing or hemolyzed serum or plasma sample at either SOALS baseline or follow-up, resulting in 598 participants whose serum markers of inflammatory (IL-6, TNF-α) and endothelial dysfunction (sICAM-1 and sVCAM-1) were measured in 2016.

**Figure 2:**
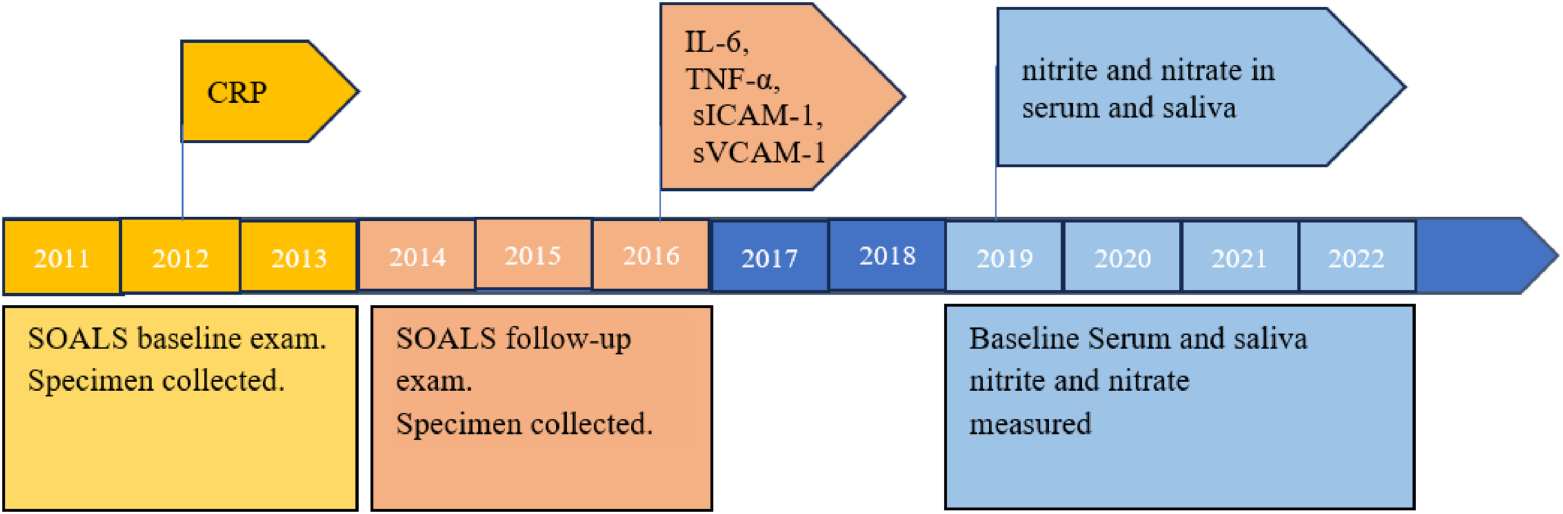
Measurement timeline of biomarkers, nitrite and nitrate.

In summary, after replacing one extreme value for salivary nitrate, serum IL-6, TNF-α, sICAM-1, and sVCAM-1 with missing value, the final sample size for this study included the following measurements: serum nitrite and nitrate (*n* = 1,064), salivary nitrite (*n* = 1,078), salivary nitrate (*n* = 1,077), serum IL-6, TNF-α, sICAM-1, sVCAM-1 (*n* = 597) and hs-CRP (*n* = 1,197) (Figures 1, 2).

Using the G* Power software, at an alpha level of 0.05, a sample size of 597 enables us to evaluate correlations as low as 0.11 (a small effect size) with 80% power, and with a sample size of 1,000, this power even increases to 94%.

### 2.5 Analyses methods

Continuous variables were summarized as mean and standard deviation (SD), and count data were displayed as percentage. However, markers of inflammation (IL-6, TNF-α, hs-CRP) and endothelial function (sICAM-1, sVCAM-1), and nitrite and nitrate in serum and saliva were greater than zero, positively skewed and over-dispersed distributed. Their measurements were summarized as median and inter-quartile range (IQR) (Table 1). Accordingly, we used Gamma regression with robust variance estimates and log-link function to assess the associations of these biomarkers with mouthwash use and with nitrite and nitrate in serum and saliva (Tables 2, 3). Adjustments were made for major potential confounders (age, sex, smoking status, and physical activity) identified based on the literature (29–32). Physical activity was used as a confounder because physical activity plays a particularly important role in immune health during aging and is known to alter the modulation and production of nitrite oxide and pro-inflammatory cytokines, including C-reactive protein (CRP) (29). Biomarkers as well as serum and salivary nitrites and nitrates were descriptively summarized based on frequency groups of mouthwash use, and measurements were compared between groups using Wilcoxon rank-sum test (Table 4). Spearman correlations and partial correlations adjusted for covariates were calculated between biomarkers and nitrite and nitrate (Table 5).

**Table 1:** Participants’ characteristics SOALS baseline (*N* = 1,200): mean (SD) or %.

	Mean (SD) or%	Median ± IQR
Age (years)	50.4 (6.7)	—
Female %	72.8	—
Education %		—
<High school	12.2	—
High school	25.3	—
≥Some college	62.5	—
Income <$20,000 %	56.0	—
Waist circumference (cm)	106.4 (14.3)	—
Physical activity: METs	21.1 (38.4)	—
Weight (kg)	88.1 (19.5)	—
BMI	33.3 (6.3)	—
Hypertension status %		—
Normal	23.2	—
Pre-hypertension	31.6	—
Hypertension	45.2	—
Diabetes status %		—
Normal glycemia	31.2	—
Pre-diabetes	68.8	—
Current smoker %	19.1	—
Alcohol consumption (g/d)	2.3 (5.8)	—
Mouthwash Use %		—
<Once a day or never	56.1	—
Once a day	21.1	—
≥twice a day	22.8	—
hs-CRP (mg/L) (*n* = 1,197)	5.7 (6.3)	4.0 (6.4)
IL-6 (pg/ml) (*n* = 597)	1.0 (0.9)	0.8 (0.7)
TNF-a (pg/ml) (*n* = 597)	2.5 (0.9)	2.3 (0.8)
sICAM-1 (ng/ml) (*n* = 597)	539.0 (135.7)	519.4 (145.4)
sVCAM-1 (ng/ml) (*n* = 597)	599.7 (156.0)	577.2 (174.4)
Serum nitrite (µM) (*n* = 1,064)	0.079 (0.202)	0.029 (0.067)
Serum nitrate (mM) (*n* = 1,064)	0.022 (0.017)	0.019 (0.012)
Saliva nitrite (mM) (*n* = 1,078)	0.063 (0.060)	0.047 (0.044)
Saliva nitrate (mM) (*n* = 1,077)	0.140 (0.147)	0.101 (0.128)

**Table 2:** Gamma regression evaluating associations of markers of inflammation, endothelial function and nitric oxide metabolism^b^ (outcomes) with mouthwash use (exposure; ≥2/day vs. less frequent use or no use).

Outcomes	Unadjusted				Adjusted^a^				
	*N*	β	95% CI	*p* value	*N*	β	95% CI	*p* value	Significant covariates^c^
CRP	1,196	−0.002	−0.148, 0.143	0.975	1,193	−0.014	−0.157, 0.128	0.843	Age, sex, METs
IL-6	597	0.009	−0.191, 0.209	0.930	596	0.019	−0.170, 0.209	0.842	Smoke, METs
TNF-α	597	0.060	−0.011, 0.131	0.097	596	0.068	−0.002, 0.138	0.058	METs
sICAM-1	596	0.027	−0.022, 0.075	0.279	595	0.019	−0.027, 0.065	0.420	Smoke
sVCAM-1	597	0.030	−0.021, 0.082	0.248	596	0.034	−0.017, 0.085	0.198	Age
Serum nitrite	1,063	−0.35	−0.706, 0.005	0.053	1,060	−0.357	−0.650, −0.064	0.017*	Age, METs
Serum nitrate	1,063	0.025	−0.102, 0.153	0.696	1,060	0.030	−0.094, 0.153	0.638	Age, smoke
Saliva nitrite	1,077	0.098	−0.042, 0.238	0.171	1,074	0.082	−0.044, 0.208	0.203	Age, sex, smoke
Saliva nitrate	1,076	0.154	−0.005, 0.314	0.058	1,073	0.152	−0.003, 0.306	0.055	Age

^a^ Adjusted by age, sex, smoking status, and METs.

^b^ Serum nitrite in µM; serum nitrate and saliva nitrite and nitrate in mM.

^c^ Those covariates were shown significant in the adjusted models with *p* value less than 0.05.

* Statistically significant at *p* < 0.05.

**Table 3:** Gamma regression (unadjusted and adjusted^a^) models evaluating associations between markers of inflammation and endothelial function (outcome) with serum/saliva nitrite/nitrate levels^b^ (exposures; continuous measurements).

	Serum nitrite			Serum nitrate			Saliva nitrite			Saliva nitrate		
	β	*p* value	95% CI	β	*p* value	95% CI	β	*p* value	95% CI	β	*p* value	95% CI
CRP												
Unadjusted	0.06	0.533	−0.14, 0.26	−4.08	0.059	−8.31, 0.15	−0.36	0.555	−1.56, 0.84	0.05	0.832	−0.45, 0.56
Adjusted	0.09	0.397	−0.12, 0.31	−2.22	0.318	−6.60, 2.15	0.38	0.519	−0.78, 1.54	0.20	0.403	−0.27, 0.67
	Significant covariates: age, sex, physical activity (METs).											
IL-6												
Unadjusted	0.04	0.892	−0.51, 0.58	0.29	0.898	−4.08, 4.65	−0.24	0.729	−1.61, 1.12	0.14	0.650	−0.48, 0.77
Adjusted	−0.03	0.909	−0.49, 0.43	0.08	0.965	−3.63, 3.8	−0.21	0.740	−1.46, 1.03	0.07	0.806	−0.49, 0.64
	Significant covariates: smoking status (for serum NO_x_ only), physical activity (METs).											
TNF-α												
Unadjusted	−0.28*	<0.001	−0.41, −0.16	2.80*	0.001	1.22, 4.39	0.81*	0.007	0.22, 1.40	0.21	0.051	−0.0005, 0.41
Adjusted	−0.31*	<0.001	−0.43, −0.19	2.55*	0.002	0.95, 4.15	0.70*	0.022	0.10, 1.30	0.18	0.081	−0.02, 0.38
	Significant covariates: physical activity (METs) (for serum NO_x_ only)											
sICAM−1												
Unadjusted	−0.21*	<0.001	−0.29, −0.12	0.59	0.170	−0.25, 1.43	−0.04	0.825	−0.41, 0.33	0.03	0.701	−0.10, 0.16
Adjusted	−0.17*	<0.001	−0.26, −0.08	0.49	0.255	−0.36, 1.35	−0.006	0.974	−0.38, 0.37	0.07	0.330	−0.07, 0.20
	Significant covariates: smoking status.											
sVCAM-1												
Unadjusted	−0.03	0.559	−0.12, 0.06	1.76*	<0.001	0.89, 2.62	0.37	0.066	−0.02, 0.76	0.24*	0.001	0.10, 0.38
Adjusted	−0.05	0.296	−0.14, 0.04	1.56*	<0.001	0.71, 2.41	0.26	0.193	−0.13, 0.64	0.22*	0.003	0.07, 0.36
	Significant covariates: age.											

^a^ Adjusted by age, sex, smoking status, METs and frequency of mouthwash use (2 categories).

^b^ Serum nitrite in µM; serum nitrate and saliva nitrite and nitrate in mM.

* Statistically significant at *p* < 0.05.

**Table 4:** Biomarkers of inflammation, endothelial function and NO metabolism^a^ by mouthwash use frequency.

Biomarkers and NO metabolites	Mouthwash use						*p* value^b^
	Infrequent			Frequent			
	*N*	Median	IQR	*N*	Median	IQR	
CRP	924	3.96	6.44	272	4.05	6.43	0.632
IL-6	472	0.78	0.73	125	0.87	0.55	0.279
TNF-α	472	2.28	0.78	125	2.48	0.88	0.102
sICAM-1	473	517.6	144.7	123	535.7	164.6	0.271
sVCAM-1	473	575.4	173.9	124	588.5	186.4	0.261
Serum nitrite	818	0.032	0.071	245	0.023	0.045	0.003*
Serum nitrate	818	0.019	0.013	245	0.018	0.012	0.859
Saliva nitrite	849	0.045	0.043	228	0.051	0.052	0.090
Saliva nitrate	849	0.099	0.121	227	0.111	0.152	0.071

^a^ Serum nitrite in µM; serum nitrate and saliva nitrite and nitrate in mM.

^b^ *p* values of Wilcoxon rank-sum test.

* Statistically significant at *p* < 0.05.

**Table 5:** Overall spearman correlations (rho) and correlations by mouthwash use (three levels) of inflammatory and endothelial biomarkers with nitrite and nitrate in serum or saliva^c^, and covariates-adjusted partial correlation for overall (adjusted rho).

	Overall			Within each group of mouthwash use			
				Infrequent		Frequent	
	*N*	*ρ*_s^a^	adjusted *ρ*^b^	*N*	ρ_s^a^	*N*	ρ_s^a^
Serum nitrite							
CRP	1,061	0.012	0.025	816	0.014	244	0.014
IL-6	593	0.006	0.002	468	0.013	125	0.003
TNF-α	593	−0.097*	−0.145*	468	−0.139*	125	0.087
sICAM-1	592	−0.028	−0.123*	469	−0.065	123	0.157
sVCAM-1	593	0.080	−0.046	469	0.061	124	0.180*
Serum nitrate							
CRP	1,061	−0.041	−0.031	816	−0.024	244	−0.101
IL-6	593	−0.056	0.008	468	−0.080	125	0.021
TNF-α	593	0.036	0.100*	468	−0.022	125	0.231*
sICAM-1	592	0.004	0.007	469	−0.039	123	0.171
sVCAM-1	593	0.123*	0.097*	469	0.098*	124	0.214*
Saliva nitrite							
CRP	1,076	−0.047	0.006	847	−0.047	228	−0.052
IL-6	541	−0.038	−0.015	432	−0.059	109	0.029
TNF-α	540	0.052	0.109*	431	0.040	109	0.089
sICAM-1	539	−0.021	−0.022	432	−0.022	107	−0.025
sVCAM-1	540	0.055	0.041	432	0.066	108	−0.002
Saliva nitrate							
CRP	1,075	0.006	0.003	847	0.018	227	−0.044
IL-6	540	−0.015	0.022	432	−0.024	108	−0.004
TNF-α	539	0.055	0.092*	431	−0.014	108	0.316*
sICAM-1	538	0.025	0.032	432	0.020	106	0.044
sVCAM-1	539	0.121*	0.134*	432	0.092	107	0.215*

^a^ Spearman correlation coefficient.

^b^ Partial correlations were adjusted by age, sex, smoking status, physical activity, and mouthwash use (2 levels).

^c^ Serum nitrite in µM; serum nitrate and saliva nitrite and nitrate in mM.

* Statistically significant at *p* < 0.05.

Furthermore, stratified analyses were performed to examine the effect of mouthwash use on the association of biomarkers with serum or salivary nitrite and nitrate. Within each frequency group of mouthwash use (< twice daily or no use; ≥twice daily), Spearman correlations were calculated (Table 5) and Gamma regression modeling was performed (Table 6).

**Table 6:** Association between CRP, IL-6, TNF-α, sICAM-1 and sVCAM-1 (outcome) and serum/saliva nitrite/nitrate levels^a^ were stratified by mouthwash (M1^b^: infrequent use; M2^c^: frequent use) using covariate^d^-adjusted gamma regression: estimates of coefficients and their 95% CIs.

	Serum nitrite				Serum nitrate			Saliva nitrite				Saliva nitrate		
	*n*	β	*p* value	95% CI	β	*p* value	95% CI	*n*	β	*p* value	95% CI	β	*p* value	95% CI
CRP														
M1	813	0.13	0.275	−0.10, 0.36	−1.71	0.474	−6.38, 2.97	844	0.48	0.503	−0.92, 1.87	0.34	0.269	−0.27, 0.95
M2	244	−0.52	0.243	−1.40, 0.36	−5.20	0.295	−14.92, 4.53	228	−0.06	0.954	−2.08, 1.96	−0.17	0.519	−0.70, 0.35
IL-6														
M1	467	0.03	0.914	−0.56, 0.63	0.16	0.957	−5.54, 5.85	431	−0.23	0.774	−1.82, 1.36	0.14	0.671	−0.52, 0.80
M2	125	−0.33	0.093	−0.72, 0.06	−0.88	0.535	−3.66, 1.90	109	−0.55	0.521	−2.25, 1.14	−0.38	0.139	−0.89, 0.12
TNF-α														
M1	467	−0.35*	<0.001	−0.52, −0.18	1.76	0.200	−0.93, 4.46	430	0.47	0.221	−0.28, 1.21	0.03	0.790	−0.18, 0.24
M2	125	−0.15	0.141	−0.35, 0.05	3.36*	<0.001	2.07, 4.65	109	1.04*	0.002	0.39, 1.69	0.48*	<0.001	0.25, 0.71
sICAM-1														
M1	468	−0.21*	0.001	−0.32, −0.09	−0.34	0.622	−1.69, 1.01	431	−0.21	0.291	−0.61, 0.18	0.06	0.467	−0.11, 0.24
M2	123	−0.13	0.110	−0.29, 0.03	1.58*	<0.001	0.86, 2.29	107	0.42	0.121	−0.11, 0.95	0.05	0.538	−0.11, 0.22
sVCAM-1														
M1	468	−0.06	0.198	−0.14, 0.03	1.54*	0.030	0.15, 2.92	431	0.23	0.380	−0.28, 0.73	0.20*	0.025	0.02, 0.38
M2	124	−0.05	0.758	−0.36, 0.26	1.85*	<0.001	1.10, 2.60	108	0.47	0.076	−0.05, 0.99	0.28*	0.003	0.10, 0.47

^a^ Serum nitrite in µM; serum nitrate and saliva nitrite and nitrate in mM.

^b^ M1: using gamma regression model to examine the association between biomarkers and nitrite/nitrate levels among the participants who use mouthwash less than twice a day or no use.

^c^ M2: using gamma regression model to examine the association between biomarkers and nitrite/nitrate levels among the participants who use mouthwash at least twice a day.

^d^ Both models M1 and M2 were adjusted by age, sex, smoking status, and physical activity (METs).

* Statistically significant at *p* < 0.05.

Analyses were conducted using Stata 16.1.

## 3 Results

The characteristics of the participants and descriptive statistics of the study variables are summarized in Table 1. Of all participants (*n* = 1,200), 72.8% were female; 19.1% were current smokers; 12.2% had less than a high school education; 56.0% had an annual income of less than $20,000; the mean BMI was 33.3 kg/m^2^ and the mean METs was 21.1; 45.2% had hypertension, 68.8% had pre-diabetes; and 22.8% used mouthwash twice or more times per day. The median values of the measurements for serum nitrite (µM), serum nitrate, salivary nitrite and nitrate (mM) were 0.029, 0.019, 0.047 and 0.101, respectively. Median values for biomarkers were hs-CRP (4.0 mg/L), IL-6 (0.8 pg/ml), TNF-α (2.3 pg/ml), sICAM-1 (519.4 ng/ml) and sVCAM-1 (577.2 ng/ml).

### 3.1 Association between frequency of mouthwash use and markers of inflammation, endothelial dysfunction, and NO metabolites

Compared with participants who used mouthwash once a day or less, participants who used mouthwash twice a day or more had higher median levels of markers of inflammation (CRP: 4.05 vs. 3.96; IL-6: 0.87 vs. 0.78; TNF-α: 2.48 vs. 2.28), endothelial function (sICAM-1: 535.7 vs. 517.6; sVCAM-1: 588.5 vs. 575.4), salivary nitrite (0.051 vs. 0.045) and nitrate (0.111 vs. 0.099), whereas serum nitrate levels remained essentially similar (0.018 vs. 0.019) (Table 4). None of these differences were statistically significant. On the other hand, serum nitrite levels were significantly lower in participants with frequent mouthwash use (0.023 vs. 0.032; Wilcoxon rank-sum test *p* = 0.003) (Table 4). Consistent results were observed using Gamma regression models (Table 2). In these models, serum nitrite levels were significantly and inversely associated with the frequency of mouthwash use (β = −0.36, 95% CI: −0.65, −0.06) after adjusting for age, sex, smoking status, and physical activity.

### 3.2 Association of inflammatory and endothelial biomarkers with nitrite and nitrate levels

**Figure 3:**
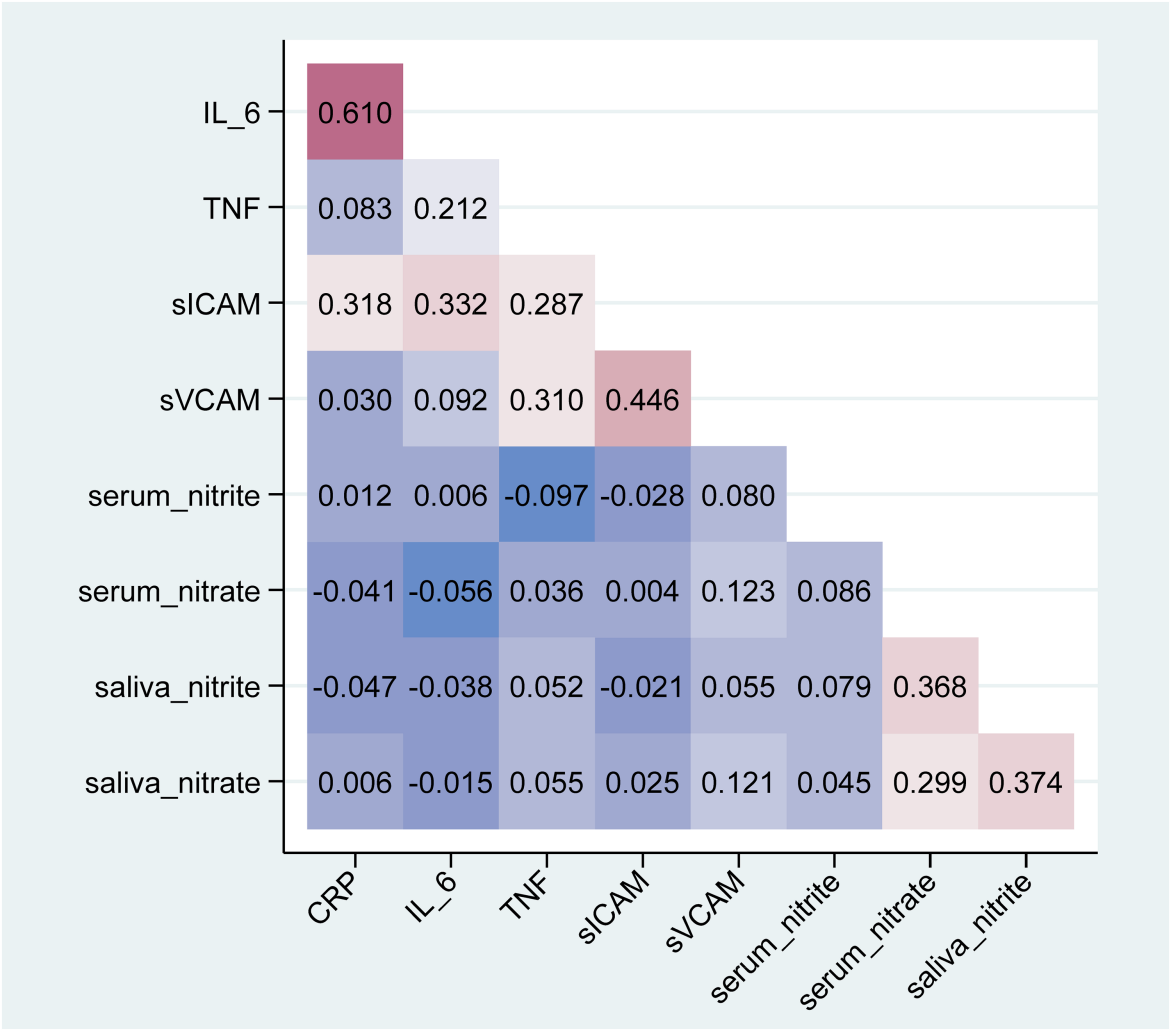
Heatmap plot of spearman pairwise correlations (rho) between inflammatory and endothelial biomarkers with nitrite and nitrate in serum or saliva.

Using Spearman correlation (Table 5, Figure 3) and Gamma regression models unadjusted or adjusted for age, sex, smoking status, physical activity and frequency of mouthwash use (Table 3), TNF-α was negatively associated with serum nitrite (unadjusted β = −0.28, 95% CI: −0.41, −0.16; adjusted β = −0.31, 95% CI: −0.43, −0.19), but positively associated with serum nitrate (unadjusted β = 2.80, 95% CI: 1.22, 4.39; adjusted β = 2.55, 95% CI: 0.95, 4.15) and salivary nitrite (unadjusted β = 0.81, 95% CI: 0.22, 1.40; adjusted β = 0.70, 95% CI: 0.10, 1.30). In addition, sVCAM-1 was positively associated with nitrate in serum (unadjusted β = 1.76, 95% CI: 0.89, 2.62; adjusted β = 1.56, 95% CI: 0.71, 2.41), and nitrate in saliva (unadjusted β = 0.24, 95% CI: 0.10, 0.38; adjusted β = 0.22, 95% CI: 0.07, 0.36). sICAM-1 was negatively associated with serum nitrite (unadjusted β = −0.21, 95% CI: −0.29, −0.12; adjusted β = −0.17, 95% CI: −0.26, −0.08). No significant associations were found between CRP and IL-6 and any serum and salivary nitrite and nitrate.

### 3.3 Impact of mouthwash on association of inflammatory and endothelial biomarkers with nitrite and nitrate levels

Spearman correlation (Table 5) and Gamma regression models adjusting for age, sex, smoking status and physical activity (Table 6) were performed to observe whether mouthwash use modulated the associations between the biomarkers and NO metabolites. In the participants who used mouthwash less than twice a day or no use, serum nitrite was negatively correlated with TNF-α (Spearman correlation *ρ*__S_ = −0.14, *p* = 0.003; Gamma regression coefficient estimate β = −0.35, 95% CI: −0.52, −0.18), and with sICAM-1 (β = −0.21, 95% CI: −0.32, −0.09). In the participants who used mouthwash twice a day or more use, TNF-α was positively associated with serum nitrate (*ρ*__S_ = 0.23, *p* = 0.010; β = 3.36, 95% CI: 2.07, 4.65), salivary nitrite (β = 1.04, 95% CI: 0.39, 1.69) and salivary nitrate (*ρ*__S_ = 0.32, *p* < 0.001; β = 0.48, 95% CI: 0.25, 0.71); in addition, sICAM-1 was positively associated with serum nitrate (β = 1.58, 95% CI: 0.86, 2.29). In both frequency groups of mouthwash users, sVCAM-1 was positively correlated with serum nitrate and salivary nitrate, and the coefficient estimates increased with the frequency of mouthwash use from 1.54 (95% CI: 0.15, 2.92) to 1.85 (95% CI: 1.10, 2.60) for serum nitrate and from 0.20 (95% CI: 0.02, 0.38) to 0.28 (95% CI: 0.10, 0.47) for salivary nitrate. In addition, sVCAM-1 was positively correlated with serum nitrite in participants who used mouthwash frequently (*ρ*__S_ = 0.18, *p* = 0.045). CRP and IL-6 were not associated with nitrite or nitrate in serum and saliva samples, either overall or within each group of mouthwash use.

## 4 Discussion

In 2020, 200 million Americans (60.2%) used mouthwash (33), and 17 million people (8.3%) used it 14 times or more in a week (34). Many mouthwash formulations contain antibacterial substances which can help reduce oral bacteria load and gingivitis (35–37), but their clinical effectiveness in preventing periodontitis and dental caries has not been proven (38, 39). The use of antibacterial mouthwashes can disrupt the oral microbiome, including beneficial bacteria involved in the production of nitric oxide (NO) through the entero-salivary pathway, and may increase the risk of cardiometabolic disease and other NO-related conditions (7, 12, 40). However, there is a paucity of research on the effects of over-the-counter mouthwash on systemic diseases. Our previous SOALS publications have shown that long-term mouthwash use may have adverse systemic effects and demonstrated a 55% increased risk for developing pre-diabetes or diabetes and an 85% increased risk of physician-diagnosed hypertension in individuals who used over-the-counter (OTC) mouthwash ≥twice a day during a 3-year follow-up period (13, 14). Unlike these previous studies that aimed to assess the association between mouthwash use and the risk of relevant clinical outcomes (e.g., hypertension, diabetes), the present study aims to assess the association between mouthwash use and nitric oxide metabolites and biomarkers, which may go some way to explaining the increased risk of hypertension and diabetes associated with mouthwash use. The results of the present study demonstrated that participants who used OTC mouthwash ≥twice a day had significantly lower systemic nitrite levels compared to less frequent mouthwash users, suggesting that the higher risk of diabetes and hypertension in these individuals could be linked to reduced NO production via the entero-salivary pathway.

Several small, short-term clinical trials have shown that mouthwash has an effect on systemic (25, 41) or salivary NO levels (26, 42, 43), or both systemic and salivary NO levels (12, 18, 44) but the effects are not consistent in mouthwashes of varying strengths and compositions (12). The majority of these trials use a prescription antibacterial mouthwash (chlorhexidine), and some of them only showed a significant immediate impact of the mouthwash on salivary nitrate and nitrite levels, rather than a systemic effect (26, 42). Few clinical trials have evaluated the impact of specific OTC mouthwashes, such as those containing essential oils or povidone-iodine, and these did not appear to significantly affect the oral nitrate reduction (45). SOALS had several major strengths compared to these studies, such as a large sample size and consideration of important confounders. In contrast to previous studies that only evaluated short-term, immediate impact of prescription-strength mouthwash, our study assessed the impact of regular, chronic use of OTC mouthwash. Our results suggest that long-term use of various types of OTC mouthwash formulations may be more significantly associated with systemic NO levels compared to the short-term use of prescription-strength mouthwashes, which appears to be primarily associated with salivary levels. Importantly, the nitrate reduction pathways of oral bacteria are rather complex and could be under the control of multiple environmental factors, such as diet, and oral hygiene practices, including mouthwash use (46). These pathways need to be better characterized in order to more accurately assess the associations between these factors and NO availability and clinical outcomes.

Nitric Oxide is a potent vasodilator and anti-inflammatory signaling molecule that plays multiple roles in the maintenance of vascular homeostasis and in inflammation (40). Under normal physiological conditions, NO acts as an anti-inflammatory mediator and inhibits the expression of TNF-α and sICAM-1. TNF-α plays a pivotal role in inflammation as a “master-regulator” of inflammatory cytokine production (47). In animal models of inflammation, dietary supplementation with nitrate acutely decreases leukocyte recruitment and reduces sICAM-1 expression in TNF-α-stimulated endothelial cells, an effect that is abolished by the use of an anti-septic mouthwash (48). These observations have suggested that NO produced by oral bacteria via the entero-salivary pathway plays the same physiological role as the endogenously produced NO in vascular endothelial function and inflammation; therefore, inhibition of this pathway by mouthwash use could negatively impact these important physiological functions (48). Consistent with these observations, TNF-α and sICAM-1 were observed to be negatively associated with serum nitrite levels in the present study; furthermore, this association was altered by mouthwash use, as it was only observed in the infrequent mouthwash users. Frequent use of OTC mouthwash was associated with higher levels of markers of inflammation (IL-6, TNF-α, CRP) and endothelial function (sICAM-1, sVCAM-1). However, the results were not statistically significant, which may reflect the localized effects of mouthwash on oral bacteria that may not significantly alter systemic inflammation markers. These markers are more likely influenced by chronic systemic conditions, which were not directly addressed in this study. The other reason may be the small sample size of 597 and the effect size detected in this study was smaller than the effect size estimated prior to the study (0.11).

Our results demonstrate that serum nitrite levels were negatively associated with inflammatory (TNF-α) and endothelial (sICAM-1) biomarker levels, especially in the participants who did not use mouthwash frequently. Consistent with these findings, frequent mouthwash use was associated with reduced serum nitrite levels and increased levels of these inflammatory and endothelial biomarkers. The relationships with other NO-metabolites (e.g., serum nitrate, and salivary nitrate and nitrite) were less significant. The reason for this may be that serum nitrite is more closely related to systemic NO bioavailability compared to the other NO metabolites in the entero-salivary pathway. The relationship between NO and other inflammatory and endothelial biomarkers was also less clear. sVCAM-1 and sICAM-1 play a central role in leukocytes recruitment, and its expression, like TNF-α, is consistently associated with and induced by active inflammation. Under certain conditions, such as in the presence of high concentrations of lipopolysaccharide, endogenously produced nitric oxide can upregulate TNF-α production in human phagocytes (49, 50). Therefore, TNF-α, as well as sVCAM-1, were positively correlated with serum nitrate and salivary nitrate. IL-6 and CRP are often viewed as inflammatory markers, but recent studies suggest that their roles are more complex. IL-6, an interleukin, is both a pro-inflammatory cytokine and an anti-inflammatory myokine, and has an inhibitory effect on TNF-α. This duality complicates the interpretation of its relationship with NO metabolites, which are primarily involved in vascular and endothelial function and immune responses (51). Unlike other biomarkers, CRP is synthesized primarily in liver hepatocytes, has a half-life of only 6–8 h, and rises rapidly and quickly returns to the normal range once treated. In addition, CRP exists in multiple isoforms, notably native CRP (nCRP) and monomeric CRP (mCRP), each of which exhibits distinct biological activities. nCRP is generally considered anti-inflammatory, while mCRP promotes inflammation. This variability may influence how CRP interacts with NO production (52). The absence of significant associations with CRP and IL-6 suggests that transient changes in NO metabolism resulting from mouthwash use may not have a measurable effect on these inflammatory markers. In addition, both CRP and IL-6 are affected by metabolic conditions (e.g., obesity and insulin resistance), which can independently affect NO metabolism (53, 54). Furthermore, their relationship may also be confounded by chronic diseases [e.g., cardiovascular disease (CVD) and diabetes] (55–57) and lifestyle factors (e.g., diet), as well as age, sex and physical activity (58, 59). In this present study, we excluded participants with known systemic conditions (e.g., CVD, diabetes, and active renal disease); age, sex and physical activity were taken into account and adjusted for; body mass index (BMI) was not included as a covariate in the analysis due to its lack of contribution to the models. Thus, it is possible that systemic inflammation driven by undiagnosed systemic conditions or even obesity may still influence the inflammatory markers observed in this study.

Due to the large number of elements included in this study (i.e., biomarkers, nitrite and nitrate in serum and saliva, mouthwash), and in order to avoid complexity and ambiguity, this present study was done cross-sectionally, focusing only on the baseline of the SOALS, despite the fact that the SOALS is longitudinal, but the disadvantage is that we were unable to make any causal inferences. In addition, we assessed the frequency of mouthwash use, particularly using at least twice a day as a threshold for frequent use, rather than the dose response, which introduced some limitations in interpreting the results. However, most OTC products are advertised to have up to 12 h of efficacy, suggesting that twice-day use could have a sustained impact on the oral microbiome. Our study used generic OTC mouthwash because the overall risk of using OTC mouthwash was the focus of SOALS’ concern and the data on mouthwash brands and ingredients were not collected. Since each brand of mouthwash contains a variety of different ingredients, different formulations may have a different impact on the nitric-oxide reducing microbiome. Although we prioritized adequate sample sizes prior to the study based on the study design and assumed small effect sizes to enhance reliability and validity, the difference in sample sizes for biomarkers (*n* = 597 for IL-6, TNF-α, sICAM-1, sVCAM-1; *n* = 1,197 for hs-CRP) and NO metabolites (*n* = 1,064 for serum; *n* = 1,077 for saliva) may have an influence on the analysis. To evaluate the potential impact of sample size differences, we conducted a sensitivity analysis, which focused on CRP and NO metabolites because their numbers of observations were larger than those of other biomarkers. In the analyses evaluating associations between NO metabolites and biomarkers (except CRP), the amount of data analyzed was made consistent across all these biomarkers (*n* = 597) by excluding automatically the participants with missing values for biomarkers (even if their NO metabolites were available). As for CRP, we re-analyzed the data using only these 597 participants and the result showed that CRP was still not significantly associated with serum nitrite, salivary nitrite and nitrate, which is in line with the results of the previous analysis, but CRP was significantly associated with serum nitrate (gamma regression models, unadjusted β = −6.78, 95% CI: −11.67, −1.89; adjusted β = −5.4, 95% CI: −10.53, −0.28), which was not previously observed when using a larger number of participants. With regard to the statistical analyses assessing the association between mouthwash use and CRP and NO metabolites, we re-ran the analyses using only these 597 participants. The results were consistent with the previous ones, except that the significant association between serum nitrite and mouthwash became nonsignificant in the adjusted gamma regression model (β = −0.23, 95% CI: −0.62, 0.16, *p* = 0.248). Thus, the difference in sample size gave us some inconsistent results. Nonetheless, we still think it is a good idea to include as many participants as possible in this study, rather than using partial participants (for CRP and NO metabolites) just to be consistent with the sample sizes of the other biomarkers (i.e., IL-6, TNF-α, sICAM-1 and sVCAM-1). On the one hand, larger samples are more accurately representative of the population and tend to average out random variability, which can improve the validity of the study and thus make it easier to detect small effects even though some of them are necessarily meaningful or practically significant; on the other hand, smaller samples might show significant results and observe inflated effect sizes due to low variability or biases, which are rather than a true effect and may not hold in larger samples (60, 61). SOALS employed standardized protocols for sample collection, processing, storage, and analysis, but as with most large epidemiological studies using frozen samples, some variation is expected; this is particularly true for nitrite in serum, which is known to have a short half-life. The laboratory measurements of nitric oxide metabolites in serum and saliva (median 7.9 years) and biomarkers (except CRP) in serum (median 4.7) were assessed a few years after collection, but these specimens had been stored at −80℃, where the effects of prolonged storage for up to 7 years at that temperature are virtually negligible (62, 63), and laboratory technicians had been strictly adhering to the storage guidelines and monitoring them. All samples were carefully inspected prior to laboratory measurements and hemolyzed serum samples were excluded. In addition, NO metabolites in saliva and serum were measured continuously by the laboratory over the same time period, eliminating possible biases or errors, and their measurements were within published ranges, demonstrating feasibility, validity, and stability of the SOALS stored samples (64, 65). Although specimens were collected at the same time point (i.e., the baseline of SOALS), biomarkers and NO metabolites were measured in the laboratory at different times. However, in our study, all one biomarker was tested during the same time interval, and in addition, strict calibration procedures and standardized methods were made to reduce the possible variability that may result from staggered laboratory measurement times. The strength of our study is the large sample size, which included more than 1,000 samples with nitric oxide metabolites and CRP measurements, as well as about 600 samples with inflammatory and endothelial biomarkers measurements. In addition, a number of important confounders were adjusted for in our analyses, including physical activity (METs) as described in the Methods.

## 5 Conclusions

We report that markers of inflammation (TNF-α) and endothelial function (sVCAM-1, sICAM-1) were associated with serum and salivary nitrite and nitrate levels in different ways, and that chronic use of over-the-counter mouthwash differentially impact the associations; furthermore, regular use of OTC mouthwash was negatively associated with serum nitrite and potentially increased levels of markers of inflammation (TNF-α) and endothelial function (sICAM-1). Our findings raise concerns about the potential impact of mouthwash on other chronic inflammatory diseases associated with endothelial dysfunction and highlight the need to investigate the impact of OTC mouthwash use on the oral microbiome, particularly on microbial pathways involved in nitric oxide metabolism.

## Data Availability

The raw data supporting the conclusions of this article will be made available by the authors, without undue reservation.
