# Supplementary material for: Association of over-the-counter mouthwash use with markers of nitric oxide metabolism, inflammation, and endothelial function—a cross-sectional study
Source: Front Oral Health. 2025 Jan 27;6:1488286. doi: 10.3389/froh.2025.1488286 (PMC11841417; doi:10.3389/froh.2025.1488286)
Supplement: Supplementary file 1 [file Datasheet1.pdf]

San Juan Overweight Adult Longitudinal Study (SOALS)

School of Dental Medicine and Graduate School of Public Health

University of Puerto Rico, Medical Sciences Campus

PERIODONTITIS AND PRECLINICAL PHASE OF DIABETES

BASELINE VISIT QUESTIONNAIRE

Code	Participant's Information	
ID1	Participant's ID number	___ ___ ___ ___
ID2	Interviewer's initials	___ ___ ___
ID3	Interview's beginning hour	___ ___ : ___ ___ AM
ID4	Interview's end hour	___ ___ : ___ ___ AM
ID5	Interview's date (DD / MM / YYYY)	___ ___ / ___ ___ / ___ ___ ___ ___

Section A: Sociodemographic Characteristics		
**Code**	**Question**	**Response**
Now we're going to start the interview. In the first section I would like to ask some general questions about you.		
A1	**Interviewer:** Indicate gender of respondent	___ (1) Female<br>___ (2) Male
A2	How old are you?	___ ___ years old
A3	What is the highest level of education you have completed?	___ (1) Never attended school / only attended kindergarten<br>___ (2) First through eighth grade<br>___ (3) Ninth to eleventh grade<br>___ (4) Twelfth or GED[1] (high school graduate)<br>___ (5) Some university or technical years (without getting a diploma)<br>___ (6) Bachelor (four years of college)<br>___ (7) Graduate work (Master's, doctoral or postdoctoral degree)<br>___ (99) Refuse

Section A: Socio-demographic Characteristics (Continuation)		
**Code**	**Question**	**Response**
A7	What is your total (gross) household income received during the last 12 months?	___ (1) < $10,000<br>___ (2) $10,000 - $19,999<br>___ (3) $20,000 - $29,999<br>___ (4) $30,000 - $39,999<br>___ (5) $40,000 - $49,999<br>___ (6) $50,000 - $59,999<br>___ (7) $60,000 - $69,999<br>___ (8) $70,000 - $99,999<br>___ (9) $100,000 or more<br>___ (99) Refuse

[1] GED=General Equivalency Diploma/General Education Development

<table>
<tr><th colspan="7">Section B: General Health Status</th></tr>
<tr><th>Code</th><th colspan="2">Question</th><th colspan="4">Response</th></tr>
<tr><td colspan="7">Now, I would like to ask some questions about your general health status.</td></tr>
<tr><td rowspan="12">B2</td><td colspan="6">Has a doctor ever diagnosed you or a first degree family member (parent or sibling) with the following conditions? (Interviewer: First, ask the participants about diagnosis and treatment of health conditions and then ask questions regarding their family history. Use codes 1=Yes, 2=No, 88=Do not know, 99=Refuse)</td></tr>
<tr><th rowspan="2">Health Condition</th><th colspan="2">Participant</th><th rowspan="2">Parent</th><th rowspan="2" colspan="2">Sibling</th></tr>
<tr><th>Diagnosis</th><th>Under pharmacological treatment</th></tr>
<tr><td>a. Coronary heart disease [evidence of blocked coronary arteries, angioplasty or coronary bypass surgery / open heart surgery or bypass]</td><td></td><td></td><td></td><td colspan="2"></td></tr>
<tr><td>b. Myocardial infarction [heart attack]</td><td></td><td></td><td></td><td colspan="2"></td></tr>
<tr><td>c. Angina pectoris [severe chest pain due to lack of oxygen of the heart muscle]</td><td></td><td></td><td></td><td colspan="2"></td></tr>
<tr><td>d. Weak or enlarged heart [heart failure, water in the lungs]</td><td></td><td></td><td></td><td colspan="2"></td></tr>
<tr><td>e. Hypertension [high blood pressure]</td><td></td><td></td><td></td><td colspan="2"></td></tr>
<tr><td>f. Hypercholesterolemia [high blood cholesterol]</td><td></td><td></td><td></td><td colspan="2"></td></tr>
<tr><td>g. High LDL cholesterol [bad cholesterol]</td><td></td><td></td><td></td><td colspan="2"></td></tr>
<tr><td>h. Reduced HDL cholesterol [good cholesterol]</td><td></td><td></td><td></td><td colspan="2"></td></tr>
<tr><td>i. Elevated triglycerides</td><td></td><td></td><td></td><td colspan="2"></td></tr>
<tr><td></td><td>o. Diabetes mellitus [type 1, type 2 or gestational diabetes]
[specify age at diagnosis: ____________]</td><td></td><td></td><td></td><td colspan="2"></td></tr>
<tr><td></td><td>p. Pre-diabetes or borderline diabetes</td><td></td><td></td><td></td><td colspan="2"></td></tr>
<tr><td></td><td>q. Chronic lung disease [emphysema or chronic bronchitis]</td><td></td><td></td><td></td><td colspan="2"></td></tr>
</table>

Section C: Oral Health Status (Continuation)								
**Code**	**Question**	**Response**						
C9	How often do you… (**Interviewer:** Mark with "X" the response.)							
		Never	Less than once a week	1-3 times a week	4-6 times a week	Once a day	Twice a day	More than twice a day
	a. brush your teeth?							
	b. floss your teeth?							
	c. use other oral hygiene aids?							
	If you use other oral hygiene aid, please specify:	______________________________						

Section D: Physical Activity													
Now, I would like to ask some questions about the time you spent at various forms of physical activity.													
**Code**	**Question**	**Response**											
D1	During the past year, what was your average total time per week at each activity?												
	**Activity**	**Average Total Time per Week**											
		**None**	**1-4 min.**	**5-19 min.**	**20-39 min.**	**40-80 min.**	**1.5 hrs.**	**2-3 hrs.**	**4-6 hrs.**	**7-10 hrs.**	**11-20 hrs.**	**21-30 hrs.**	**31-40 hrs.**
	Siting at work												
	Sitting or driving in a vehicle (car, bus, train)												
	Sitting or lying watching TV or DVD												
	Sitting or lying at home reading												
	Sitting at home working on a computer												
	Other sitting or lying at home (at desk or eating)												

Section D: Physical Activity (Continuation)													
**Code**	**Question**						**Response**						
D2	During the past year, what was your average total time per week at each activity?												
	**Activity**	**Average Total Time per Week**											
		**None**	**1-4 min.**	**5-19 min.**	**20-39 min.**	**40-80 min.**	**1.5 hrs.**	**2-3 hrs.**	**4-6 hrs.**	**7-10 hrs.**	**11-20 hrs.**	**21-30 hrs.**	**31-40 hrs.**
	Walking to work or for exercise (including golf)												
	Jogging (slower than 10 minutes/mile)												
	Running (10 minutes/mile or faster)												
	Bicycling (including stationary machine)												
	Lap swimming												
	Tennis												
	Squash or racquetball												
	Calisthenics, rowing, stair or ski machine, etc.												
	Weightlifting or weight machine												
	Moderate outdoor work (e.g., yard work, gardening)												
	Heavy outdoor work (e.g., digging, chopping)												
	Dancing												
	Other forms of physical activity												

Section E: Alcohol Consumption and Tobacco Use		
Now, I would like to ask some questions about your alcohol and tobacco use.		
**Code**	**Question**	**Response**
E1	During the past 12 months, how frequently have you had at least one alcoholic drink?	___ (1) daily<br>___ (2) 5 – 6 days / week<br>___ (3) 1 – 4 days / week<br>___ (4) 1 – 3 days / month<br>___ (5) Less than once a month<br>___ (6) Never **[Proceed to Question E5]**<br>___ (88) Do not know<br>___ (99) Refuse
E2	During the past 30 days, when you drank alcohol, on average, how many standard alcoholic drinks did you have during one drinking occasion? [**A standard drink consists of a 12-oz. bottle or can of regular beer (~5% alcohol), a 5-oz. glass of table wine (~12% alcohol), and a 1.5-oz. drink of 80 proof (~40% alcohol) distilled spirits or liquor (either straight or in a mixed drink)**].	___ ___ standard drinks<br>___ (0) Did not drink **[Proceed to Question E5]**<br>___ (88) Do not know<br>___ (99) Refuse
E3	During the past 30 days, what was the largest number of standard alcoholic drinks you had on a single occasion, counting all types of alcoholic drinks together?	___ ___ standard drinks<br>___ (88) Do not know<br>___ (99) Refuse
E4	During the past 30 days, how many times did you have ___ ___ [**Men: five or more**, **Women: four or more**] standard alcoholic drinks in a single drinking occasion?	___ ___ times<br>___ (88) Do not know<br>___ (99) Refuse

Section E: Alcohol Consumption and Tobacco Use (Continuation)		
**Code**	**Question**	**Response**
E7	Do you currently smoke cigarettes?	___ (1) Yes<br>___ (2) No **[Proceed to Question E9]**<br>___ (88) Do not know **[Proceed to Section E9]**<br>___ (99) Refuse **[Proceed to Section E9]**
E8	How many cigarettes do you smoke daily or weekly?	___ ___ cigarettes / day **[Proceed to Section E11]**<br>___ ___ cigarettes / week **[Proceed to Section E11]**<br>___ (88) Do not know **[Proceed to Section E11]**<br>___ (99) Refuse **[Proceed to Section F]**
E9	How old were you when you stopped smoking?	___ ___ years<br>___ (88) Do not know<br>___ (99) Refuse
E10	How many cigarettes per day or per week did you smoke?	___ ___ cigarettes / day<br>___ ___ cigarettes / week<br>___ (88) Do not know<br>___ (99) Refuse
E11	Do you currently use other tobacco products such as chewing tobacco, cigar or pipe every day, some days, or not at all?	___ (1) Every day<br>___ (2) Some days<br>___ (3) Not at all<br>___ (88) Do not know<br>___ (99) Refuse
