# Supplementary material for: Association of over-the-counter mouthwash use with markers of nitric oxide metabolism, inflammation, and endothelial function—a cross-sectional study
Source: Front Oral Health. 2025 Jan 27;6:1488286. doi: 10.3389/froh.2025.1488286 (PMC11841417; doi:10.3389/froh.2025.1488286)
Supplement: Supplementary file 2 [file Datasheet2.pdf]

STROBE Checklist of items that should be included in reports of cross-sectional studies with detailed referencing or requirements to the text of the paper.

	Item No	Recommendation
**Title and abstract**	1	(*a*) Indicate the study's design with a commonly used term in the title or the abstract<br>Cross-sectional study, focusing on SOALS baseline participants and specimens, as stated in the title, the Abstract on page 2 and the Methods on page 7.
		(*b*) Provide in the abstract an informative and balanced summary of what was done and what was found<br>Provided in the Abstract on page 2.
**Introduction**		
Background/rationale	2	Explain the scientific background and rationale for the investigation being reported<br>Included in the Introduction on pages 3, 4 and 5.
Objectives	3	State specific objectives, including any prespecified hypotheses<br>Included in the Introduction on pages 4 and 5.
**Methods**		
Study design	4	Present key elements of study design early in the paper<br>Included in the Study Population, Sample collection and laboratory measurements, and Mouthwash use and covariates assessment of Methods on pages 6-10.
Setting	5	Describe the setting, locations, and relevant dates, including periods of recruitment, exposure, follow-up, and data collection<br>Included in the Methods on pages 6-10, and Figure 1 and 2 on pages 27 and 28.
Participants	6	(*a*) Give the eligibility criteria, and the sources and methods of selection of participants<br>Included in the Study Population of the Methods on pages 6 and 7, and Figure 1 on page 27.
Variables	7	Clearly define all outcomes, exposures, predictors, potential confounders, and effect modifiers. Give diagnostic criteria, if applicable<br>Included in the Laboratory measurements, Mouthwash use and Covariates assessment of the Methods on pages 8-10.
Data sources/ measurement	8*	For each variable of interest, give sources of data and details of methods of assessment (measurement). Describe comparability of assessment methods if there is more than one group<br>Included in the Study Population, Laboratory measurements, Mouthwash use and Covariates assessment of the Methods on pages 6-10.
Bias	9	Describe any efforts to address potential sources of bias<br>Addressed in the Laboratory measurements of the Method on page 9, and in the limitation paragraph on pages 19 - 21 in the Discussion.
Study size	10	Explain how the study size was arrived at<br>Included in the Sample Size of the Methods on page 10 and the top of the page 11.
Quantitative variables	11	Explain how quantitative variables were handled in the analyses. If applicable, describe which groupings were chosen and why<br>Included in the Analyses Methods of the Methods on page 11.
Statistical methods	12	(*a*) Describe all statistical methods, including those used to control for confounding<br>Included in the Analyses Methods of the Methods on pages 11 and 12.
		(*b*) Describe any methods used to examine subgroups and interactions<br>Included in the Mouthwash use and Covariates assessment of the Methods on pages

		9 and 10, and the Analyses Methods of the Methods on pages 11 and 12.
		(*c*) Explain how missing data were addressed<br>Included in the Sample Size of the Methods on page10.
		(*d*) If applicable, describe analytical methods taking account of sampling strategy<br>Not applicable.
		(*e*) Describe any sensitivity analyses<br>Included in the limitation paragraph on page 20 of the Discussion for discrepancy in sample sizes.
**Results**		
Participants	13*	(a) Report numbers of individuals at each stage of study—eg numbers potentially eligible, examined for eligibility, confirmed eligible, included in the study, completing follow-up, and analysed<br>Included in the Results on page 12, and summarized in Table 1 on pages 22 and 23 and Figure 1 on page 27.
		(b) Give reasons for non-participation at each stage<br>Summarized in Figure 1 on page 27.
		(c) Consider use of a flow diagram<br>Figure 1 on page 27.
Descriptive data	14*	(a) Give characteristics of study participants (eg demographic, clinical, social) and information on exposures and potential confounders<br>Included in the Results on page 12, and summarized in Table 1 on pages 22 and 23.
		(b) Indicate number of participants with missing data for each variable of interest<br>Summarized in Table 1 on pages 22 and 23.
Outcome data	15*	Report numbers of outcome events or summary measures<br>Included in the Results on pages 12-13, and summarized in Tables 1 and 2 on pages 22 and 23.
Main results	16	(*a*) Give unadjusted estimates and, if applicable, confounder-adjusted estimates and their precision (eg, 95% confidence interval). Make clear which confounders were adjusted for and why they were included<br>Included in the Results on pages 12-14, and summarized in Tables 1-6 and Figure 3 on page 28.
		(*b*) Report category boundaries when continuous variables were categorized<br>Not applicable.
		(*c*) If relevant, consider translating estimates of relative risk into absolute risk for a meaningful time period<br>Not applicable.
Other analyses	17	Report other analyses done—eg analyses of subgroups and interactions, and A<br>Analyses by frequency of mouthwash use were included in the Results on page 14, and sensitivity analyses for discrepancy in sample sizes were included in the limitation paragraph on page 20 of the Discussion.
**Discussion**		
Key results	18	Summarise key results with reference to study objectives<br>Included in the Discussion on pages 15-19.
Limitations	19	Discuss limitations of the study, taking into account sources of potential bias or imprecision. Discuss both direction and magnitude of any potential bias<br>Included in the Discussion on pages 19-21.
Interpretation	20	Give a cautious overall interpretation of results considering objectives, limitations,

		multiplicity of analyses, results from similar studies, and other relevant evidence<br>Included in the Discussion on pages 15-19.
Generalisability	21	Discuss the generalisability (external validity) of the study results<br>Included in the Discussion on pages 15-21.
**Other information**		
Funding	22	Give the source of funding and the role of the funders for the present study and, if applicable, for the original study on which the present article is based<br>Provided in the text on page 29.

*Give information separately for exposed and unexposed groups.
